# A Systematic Review of Second-Line Treatments in Antiviral Resistant Strains of HSV-1, HSV-2, and VZV

**DOI:** 10.7759/cureus.35958

**Published:** 2023-03-09

**Authors:** Kimberly C Lince, Virgil K DeMario, George T Yang, Rita T Tran, Daniel T Nguyen, Jacob N Sanderson, Rachel Pittman, Rebecca L Sanchez

**Affiliations:** 1 Department of Clinically Applied Science Education, University of the Incarnate Word School of Osteopathic Medicine, San Antonio, USA

**Keywords:** vzv, hsv-2, anti-hsv, second line drugs, resistant hsv, experimental pharmacology, general obgyn, antiviral resistance, direct anti-viral agents, hsv-1

## Abstract

Drug-resistant variants of herpes simplex viruses (HSV) have been reported that are not effectively treated with first-line antiviral agents. The objective of this study was to evaluate available literature on the possible efficacy of second-line treatments in HSV and the use of second-line treatments in HSV strains that are resistant to first-line treatments. Following Preferred Reporting Items for Systematic Reviews and Meta-Analyses (PRISMA) guidelines, a final search was conducted in six databases on November 5, 2021 for all relevant literature using terms related to antiviral resistance, herpes, and HSV. Eligible manuscripts were required to report the presence of an existing or proposed second-line treatment for HSV-1, HSV-2, or varicella zoster virus (VZV); have full-text English-language access; and potentially reduce the rate of antiviral resistance.

Following screening, 137 articles were included in qualitative synthesis. Of the included studies, articles that examined the relationship between viral resistance to first-line treatments and potential second-line treatments in HSV were included. The Cochrane risk-of-bias tool for randomized trials was used to assess risk of bias. Due to the heterogeneity of study designs, a meta-analysis of the studies was not performed. The dates in which accepted studies were published spanned from 2015-2021. In terms of sample characteristics, the majority (72.26%) of studies used Vero cells. When looking at the viruses on which the interventions were tested, the majority (84.67%) used HSV-1, with (34.31%) of these studies reporting testing on resistant HSV strains. Regarding the effectiveness of the proposed interventions, 91.97% were effective as potential managements for resistant strains of HSV. Of the papers reviewed, nectin in 2.19% of the reviews had efficacy as a second-line treatments in HSV, amenamevir in 2.19%, methanol extract in 2.19%, monoclonal antibodies in 1.46%, arbidol in 1.46%, siRNA swarms in 1.46%, Cucumis melo sulfated pectin in 1.46%, and components from Olea europeae in 1.46%. In addition to this griffithsin in 1.46% was effective, Morus alba L. in 1.46%, using nucleosides in 1.46%, botryosphaeran in 1.46%, monoterpenes in 1.46%, almond skin extracts in 1.46%, bortezomib in 1.46%, flavonoid compounds in 1.46%, andessential oils were effective in 1.46%, but not effective in 0.73%.

The available literature reviewed consistently supports the existence and potentiality of second-line treatments for HSV strains that are resistant to first-line treatments. Immunocompromised patients have been noted to be the population most often affected by drug-resistant variants of HSV. Subsequently, we found that HSV infections in this patient population are challenging to manage clinically effectively. The goal of this systematic review is to provide additional information to patients on the potentiality of second-line treatment in HSV strains resistant to first-line treatments, especially those who are immunocompromised. All patients, whether they are immunocompromised or not, deserve to have their infections clinically managed in a manner supported by comprehensive research. This review provides necessary information about treatment options for patients with resistant HSV infections and their providers.

## Introduction and background

Although antiviral agents, including acyclovir, ganciclovir, and foscarnet, hold a vital role in the clinical management of herpes virus infections, drug-resistant variants of herpes simplex viruses (HSV) have been reported that are not effectively treated with these drugs [1,2]. Immunocompromised patients have been reported to be the primary population to present with viral strains that have mutations conferring resistance [1,3]. The primary goal of this systematic review is to evaluate the available literature on the possible efficacy of second-line treatments in HSV strains that are resistant to first-line treatments.

## Review

Material and methods

Following prospectively registered protocol and Preferred Reporting Items for Systematic Reviews and Meta-Analyses (PRISMA) guidelines [4], a final search was conducted in six databases including PubMed via PubMed.gov, Embase via Embase.com, the Cochrane Library via Wiley, Web of Science, Clinicaltrials.gov, and ScienceDirect on November 5, 2021, for all relevant literature using terms including antiviral resistance, herpes virus, and HSV. Two authors independently reviewed studies for adherence to criteria, conducted risk assessments, and extracted results.

Eligibility Criteria

**Figure 1:**
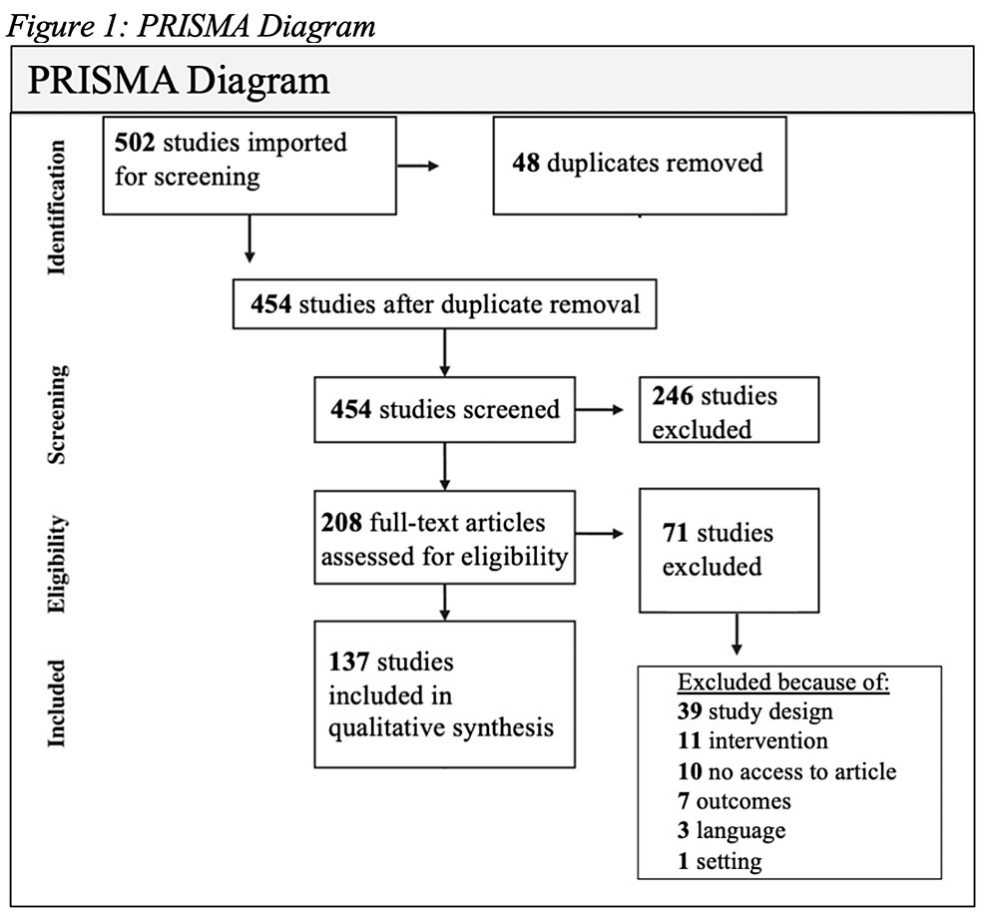
PRISMA diagram PRISMA: Preferred Reporting Items for Systematic Reviews and Meta-Analyses

Eligible manuscripts were required to report the presence of an existing or proposed second-line treatment for HSV-1, HSV-2, or varicella zoster virus (VZV) and have full-text English-language access. Types of studies that were included within the search encompassed randomized controlled trials, research studies or articles, and research reports including alternative grey literature (Figure 1). Systematic reviews, clinical practice guidelines, and case reports were excluded. There were no limitations applied to this study that involved gender and age. The Cochrane risk-of-bias tool for randomized trials was used to assess the risk of bias. The study team conducted a database search of six databases on November 5, 2021.This search did not have severe limitations and was broadly inclusive in terms of types of articles and antiviral approaches. A broad search was conducted that included key terminology such as “antiviral resistance”, “herpes virus”, and “HSV” and was limited to English language studies published after 2015.

Study Selection

Utilizing the Covidence systematic review manager, two individual reviewers independently analyzed each publication for inclusion or exclusion criteria. Discrepancies were resolved through discussion by the entire review team including two subject matter experts.

Data Extraction

Seven reviewers independently analyzed manuscripts in a two-part methodology that consisted of a first pass and second pass so that each article was reviewed twice by two individual reviewers. In both the first and second pass, each reviewer extracted standardized information in accordance with the study objective. This process reported details such as a confirmation of intervention existence as well as details including intervention description, if the intervention was tested on HSV-1, HSV-2, and/or VZV and if said intervention was tested on a resistant strain of the virus.

Reviewers also outlined the methodology of the study as well as extracted what mutation conferring resistance was present (if any) and efficacy of the intervention. Reviewers stated the type of study in each article, sample size, sample characteristics, and if the intervention was recommended for inclusion or exclusion. Discrepancies were resolved through discussion by the entire review team including two subject matter experts. The main outcomes evaluated were the efficacy of a novel intervention against HSV-1, HSV-2, and VZV and the efficacy of these antivirals against drug-resistant variants of herpes simplex viruses. Efficacy was primarily determined by a decline in viral load, severity of infection, and/or reported interference with viral replication.

Assessment of Risk of Bias

The Cochrane Risk of Bias Tool for randomized trials was used to assess risk of bias including hidden allocations for personnel and participants [5]. There was also an evaluation of the generation of sequences in addition to outcome blindness, outcome data that was incomplete, reporting that was deemed selective, and additional sources of bias. The reporting, statistical results, and conflicts of interest, including funding, were also analyzed in terms of efficacy for drug-resistant variants of herpes simplex viruses for each proposed intervention.

Independently, two reviewers analyzed each of the 137 included studies, classifying them as ‘high’, ‘low’, or ‘unclear’ risk of bias for each criterion. A third reviewer resolved discrepancies in quality assessment. For each domain, the reasoning for the decision was detailed. The full-text manuscripts for individual studies were utilized as a summary of the reason for the decision. Among other domains included in this quality assessment, ‘other sources of bias’ were not included in this quality assessment. 

Data Synthesis

Due to the heterogeneity of study designs, a meta-analysis of the studies was excluded. Pertinent study details were instead narrated.

Results

Study Selection

The initial literature search yielded 597 references. After duplicate removal, 454 articles underwent title and abstract screening. This yielded 208 articles eligible for full-text review that resulted in 137 articles that were included in qualitative synthesis.

Data Extraction

Of the included studies, articles that examined the relationship between viral resistance to first-line treatments and potential second-line treatments in HSV were included. A meta-analysis of the studies was excluded because of the nature of the study.

Synthesis of Results

The dates in which accepted studies were published spanned from 2015-2021. Within this time frame, there were two (1.46%) studies in 2015 [6,7], 15 (10.95%) studies in 2016 [1,3,8-20], 19 (13.87%) studies in 2017 [21-39], 27 (19.71%) studies in 2018 [40-64], 23 (16.79%) studies in 2019 [2,65-86], 24 (17.52%) studies in 2020 [87-110], and 27 (19.70%) studies in 2021 [111-137]. Regarding the type of studies reviewed, 121 (88.32%) studies included in vitro elements [2,3,6-12,14-20,23-26,28-42,44-47,49-56,58-64,66,68-72,74-77,79,80,82-87,89-118,120,122-133,135-139] and 23 (16.79%) in vivo [2,13,15,18,22,25,27,28,37,43,48,56,62,67,73,78,81,100,106,108-110,134,137]. Sample size was extremely variable and unable to be compared due to the differences in study designs. In terms of sample characteristics, 99 (72.26%) studies used Vero cells [2,3,6,8-10,14-20,23,25,26,28,30-34,36,39-42,44-47,50,51,53-56,58-64,66-70,72,74,76,77,79,82,83,85-87,89-95,97-108,111-113,115-118,120,122,123,125-130,132,133,135,137,138], 41 (29.93%) used human cell lines [7,11,12,16,19,24,29,31,36,38,47,49,52-56,61,62,67,69,71,75,78,80,83,84,96,99,100,102,104,109,110,112,114, 115,124,130,135,136], and 12 (8.76%) used various other animal cells (not Vero) [7,19,35,37,64, 78,86,89,109,126,131,139]. Of the 23 in vivo studies, mice and guinea pigs were the most common model [2,13,15,18,22,25,27,28,37,43,56,62,64,73,81,100,106,108-110,134,137].

Regarding which viruses the interventions were tested on, 113 (82.48%) studies used HSV-1 [1-3,6-16,19,21-27,29-31,33-35,37,38,40-44,46-49,51,53-56,60-65,67-74,76-79,82,83,85-99,103-123,125-134,136-138], 61 (44.53%) studies used HSV-2 [7-11,14-20,25,27,28,30-33,35-37,39,44-53,55,58,59,61, 66,69,73,75,81,83,85,89,91,93,100,102,104,105, 108-110,118,124,129,135-138], 7 (5.11%) used VZV [21,25,38,43,48,60,121] and 47 (34.31%) of these studies [1,2,6,9,11,13-15,19,20,27,30,31,33,35,38,39, 42,47,49,61,67,69,76,83,89-91,93,96,98,104,106,110,113,115,116,120,121, 123,126,128,130,131,134,136,137] reported testing on resistant HSV strains.

When determining the effectiveness of the proposed interventions, five (3.65%) were shown not to be effective [84,107,126,131,139], three (2.19%) were somewhat effective [21,46,53], and 128 (93.43%) were effective as potential managements for resistant strains of HSV [1-3,6-20,22-45,47-52,54-56,58-83,85-106,108-125,127-130,132-138].

Of the papers reviewed, three (2.19%) papers displayed that nectin had efficacy as a second-line treatments in HSV [17,28,73], three (2.19%) papers showed efficacy using amenamevir [21,43,95], three (2.19%) using methanol extract were effective in various cell line [24,32,39], two (1.46%) using monoclonal antibodies [27,46], two (1.46%) with arbidol [75,81], two (1.46%) with siRNA swarms [22,98], two (1.46%) using Cucumis melo sulfated pectin were effective in Vero cells [10,123], two (1.46%) using components from Olea europeae were effective in Vero cells [125,130]. In addition to this, two (1.46%) with griffithsin (GRFT) were effective in various cell lines [45,80], two (1.46%) with Morus alba L. (compounds from mulberry root bark extract) were effective in Vero cells [85,103], two (1.46%) using nucleosides were effective in Vero cells [41,76], two (1.46%) with botryosphaeran were effective in Vero cells [74,128], two (1.46%) monoterpenes were effective in Vero cells [34,92], two (1.46%) with almond skin extracts were effective in Vero cells [26,68], two (1.46%) using bortezomib were effective in HCL and Vero cells [83,99], two (1.46%) using various flavonoid compounds [25,97], and two (1.46%) papers using essential oils were effective in Vero cells [31,34], but one (0.73%) showed that essential oils were not effective [131]. 

Main characteristics of studies included in the systematic review is shown in Table 1.

**Table 1:** Main characteristics of studies included in the systematic review

Main characteristics of studies included in the systematic review													
First author	Title	Year	Type of study	Sample characteristics	Intervention	Intervention description	Tested on HSV-1?	Tested on HSV-2?	Tested on VZV?	Tested on resistant strain(s)?	Mutation conferring resistance	Efficacy?	Citation
Agostinho	Cucumis melo pectin as potential candidate to control herpes simplex virus infection	2021	In-vitro	Vero cells	Y	Cucumis melo sulfated pectin	Y	N	N	Y	ACV resistant	Y	[123]
Al-Salahi	Molecular docking study and antiviral evaluation of2-thioxo-benzo[g]quinazolin-4(3H)-one derivatives	2016	In-vitro	Vero cells	Y	Cucumis melo sulfated pectin	Y	Y	N	N	N/A	Y	[10]
Alvarez	Cetylpyridinium chloride blocks herpes simplex virus replication in gingival fibroblasts	2020	In-vitro	Epithelial cells, primary human gingival fibroblasts, Vero cells	Y	Cetylpyridinium chloride (CPC)	Y	Y	N	Y	ACV resistant	Y	[104]
Andrei	The Anti-Human Immunodeficiency Virus Drug Tenofovir, a Reverse Transcriptase Inhibitor, Also Targets the Herpes Simplex Virus DNA Polymerase	2018	In-vitro	Human embryonic lung (HEL) fibroblasts	Y	HSV-1 and HSV-2 mutants that are resistance to tenofovir and PMEO-DAPy were retested with PMEO-DAPy	Y	Y	N	Y	Tenofovir and PMEO-DAPy–resistant	Y	[49]
Andronova	Study of Antiherpetic Efficiency of Phosphite of Acycloguanosine Able to Overcome the Barrier of Resistance to Acyclovir	2016	In-vivo	Male white mice, male Agouti guinea pigs	Y	Phosphite of acycloguanosine	Y	N	N	Y	ACV resistant HSV-1	Y	[13]
Arunkumar	Study on antiviral activities, drug-likeness and molecular docking of bioactive compounds of Punica granatum L. to Herpes simplex virus - 2(HSV-2)	2018	In-vitro	Human Epidermoid larynx carcinoma cell line	Y	Punica granatum fruit	N	Y	N	N	N/A	Y	[52]
Awad	Synthesis and Evaluation of Some Uracil Nucleosides as Promising Anti-Herpes Simplex Virus 1 Agents	2021	In-vitro	Vero cells	Y	In-vitro cyclic and acyclic nucleosies that incorporated 6-substituted- pyrimidine moieties	Y	N	N	N	N/A	Y	[111]
Barboza	In vitro effects of bufotenine against RNA and DNA viruses	2021	In-vitro	N/A	Y	Bufotenine, an alkaloid that can be found in plant extracts and skin secretions of amphibians	Y	N/A	N	Y	ACV resistant HSV-1	N	[126]
Bauer	Antibody-based immunotherapy of acyclovir resistant ocular herpes simplex virus infections	2017	In-vivo	BALB/c mice	Y	Humanized monoclonal antibody (mAb) hu2c that targeted the HSV-1/2 glycoprotein B	Y	Y	N	Y	ACV resistant	Y	[27]
Ben-Amor	Phytochemical Characterization of Olea europea Leaf Extracts and Assessment of Their Anti-Microbial and Anti-HSV-1 Activity	2021	In-vitro	Vero cells	Y	Leaf extracts obtained from Olea europea L. var. sativa (OESA) and Olea europea var. sylvestris (OESY) from Tunisia	Y	N	N	N	N/A	Y	[125]
Benassi-Zanqueta	Evaluation of anti-HSV-1 activity and toxicity of hydroethanolic extractof Tanacetum parthenium (L.) Sch.Bip. (Asteraceae)	2018	In-vitro	N/A	Y	Crude extract of aerial parts of Tanacetum parthenium (L.) Sch.Bip. (Asteraceae), Liquid chromatography-mass spectrometry	Y	N	N	N	N/A	Y	[64]
Benzekri	Anti HSV-2 activity of Peganum harmala (L.) and isolation of the active compound	2017	In-vitro	Vero cells	Y	Methanol Seeds extract, know as alled Peganum harmala	N	Y	N	N	N/A	Y	[32]
Bereczki	Synthesis of Antiviral Perfluoroalkyl Derivatives of Teicoplanin and Vancomycin	2020	In-vitro	N/A	Y	Teichoplanins, a glycopeptide antibiotic derivative bearing perfluroroalkyl side chains	Y	Y	N	Y	TK mutation	Y	[89]
Bhutta	Peptide Inhibitor of Complement C1, RLS-0071, Reduces Zosteriform Spread of Herpes Simplex Virus Type 1 Skin Infection and Promotes Survival in Infected Mice	2021	In-vivo	BALB/cJ Mice	Y	RLS-0071, also known as peptide inhibitor of complement C1 (PIC1)	Y	N	N	Y	ACV resistant HSV-1	Y	[134]
Bhutta	Ginkgolic Acid Inhibits Herpes Simplex Virus Type 1 Skin Infection and Prevents Zosteriform Spread in Mice	2021	In-vitro	Vero cells	Y	Ginkgolic acid	Y	N	N	Y	ACV resistant HSV-1	Y	[120]
Bisignano	Almond Skin Extracts Abrogate HSV-1 Replication by Blocking Virus Binding to the Cell	2017	In-vitro, experimental study	Vero cells	Y	Extracts with the prevalent compounds quercetin, epicatechin and catechin	Y	N	N	N	N/A	Y	[26]
Bonvicini	Hemidesmus indicus (L.) R. Br. extract inhibits the early step of herpes simplex type 1 and type 2 replication	2018	In-vitro, experimental study	Vero cells	Y	A hydroalcoholic extract from Hemidesmus indicus root	Y	Y	N	N	N/A	Y	[44]
Brenner	The Molecular Tweezer CLR01 Inhibits Antibody-Resistant Cell-to-Cell Spread of Human Cytomegalovirus	2021	In-vitro, experimental study	Human foreskin fibroblasts (HFF)	Y	CLR01	Y	Y	N	Y	Multi-resistant HSV-2	Y	[136]
Brezáni	Anti-Infectivity against Herpes Simplex Virus and Selected Microbes and Anti-Inflammatory Activities of Compounds Isolated from Eucalyptus globulus Labill	2018	In-vitro	Vero cells	Y	12 pure compounds and one mixture of two constitutional isomers from the leaves and twigs of E. globulus. E. Golulus from the Centrum of Medicinal Plants of the Medical Faculty of Masaryk University in Brno	Y	Y	N	N	N/A	Y	[138]
Cagno	In vitro anti-herpes simplex virus-2 activity of Salvia desoleana Atzei & V. Picci essential oil	2017	In-vitro	Vero cells, epithelial cell lines Hep-2	Y	S. desoleana EO, fractions and main components: linalyl acetate, alpha terpinyl acetate, and germacrene D	Y	Y	N	Y	ACV resistant HSV-2	Y	[31]
Castillo	Anti-herpetic Activity of Macrocystis pyrifera and Durvillaea antarctica Algae Extracts Against HSV-1 and HSV-2	2020	In-vitro, in-vivo	Human cervix epithelial cell line (HeLa cells), primary human gingival fibroblasts, a mouse model	Y	Aqueous extracts obtained from two brown macroalgae, namely Macrocystis pyrifera and Durvillaea antarctica	Y	Y	N	Y	ACV resistant HSV-1	Y	[110]
Chen	Targeting Aryl Hydrocarbon Receptor Signaling Enhances Type I Interferon-Independent Resistance to Herpes Simplex Virus	2021	In-vitro	Human monocytic THP-1 cells, human foreskin fibroblast 1 (HFF-1) cells, Vero cells	Y	Aryl hydrocarbon receptor (AHR) signaling	Y	N	N	N	N/A	Y	[112]
Crameri	MxB is an interferon-induced restriction factor of human herpesviruses	2018	In-vitro	Glioblastoma cells, human lung adenocarcinoma cells, vero cells, HEK-293 cells, and HeLa cells	Y	MxB, protein coded for/released in response to activation of the IFN system	Y	Y	N	N	N/A	PY	[53]
Criscuolo	Synergy evaluation of anti-Herpes Simplex Virus type 1 and 2 compounds acting on different steps of virus life cycle	2018	In-vitro	Vero cells	Y	Pairing viral DNA inhibitors + human IgG mAb	Y	Y	N	N	N/A	PY	[46]
Čulenová	Multiple In vitro biological effects of phenolic compounds from Morus alba root bark	2019	In-vitro	Vero cells	Y	Morus alba L. (compounds from mulberry root bark extract)	Y	Y	N	N	N/A	Y	[85]
D'Aiuto	R430: A potent inhibitor of DNA and RNA viruses	2018	In-vitro	Vero cells and human induced pluripotent stem cells (hiPSC-neurons)	Y	Transdihydrolycoricidine (R430), a lycorane-type alkaloid derivative	Y	Y	N	Y	ACV resistant	Y	[47]
Dai	Antiviral Effect of Retro-2.1 against Herpes Simplex Virus Type 2 In Vitro	2018	In-vitro	Vero cells	Y	Retro-2.1, an optimized, more potent derivative of Retro-2cyc	N	Y	N	N	N/A	Y	[50]
Dai	Antiviral Effects of ABMA against Herpes Simplex Virus Type 2 In Vitro and In Vivo	2018	In-vitro	Vero cells	Y	The small molecule ABMA [1-adamantyl (5-bromo-2-methoxybenzyl) amine], acting on host-endosomal trafficking	N	Y	N	N	N/A	Y	[58]
Deback	Antiviral effects of Cacicol (®), a heparan sulfate biomimetic for corneal regeneration therapy, for herpes simplex virus type-1 and varicella zoster virus infection	2018	In-vitro	Vero cells	Y	Cacicol a poly-carboxymethylglucose sulfate solution that is a regernating matrix therapy agent intended for wound healing	Y	N	Y	N	N/A	Y	[60]
Derby	Griffithsin carrageenan fast dissolving inserts prevent SHIV HSV-2 and HPV infections in vivo	2018	In-vitro	Vero cells	Y	GRFT Gel for vaginal use	N	Y	N	N	N/A	Y	[45]
Deschamps	Discovery of Small-Molecule Inhibitors Targeting the E3 Ubiquitin Ligase Activity of the Herpes Simplex Virus 1 ICP0 Protein Using an In Vitro High-Throughput Screening Assay	2019	In-vitro	Vero cells	Y	ICP0, a promiscuous transactivator that enables viral gene expression by disrupting DNA repressor complexes and blocking antiviral responses	Y	N	N	N	N/A	Y	[82]
Ding	T-type calcium channels blockers inhibit HSV-2 infection at the late stage of genome replication	2020	In-vitro	Vero cells, HeLa Cells	Y	T-type calcium channel blockers	N	Y	N	N	N/A	Y	[102]
Ding	Cellular Signaling Analysis shows antiviral, ribavirin-mediated ribosomal signaling modulation	2019	In-vitro	Vero cells	Y	Ribavirin-mediated ribosomal signaling modulation, interferons, and S6 kinase inhibitor SL010	Y	N	N	N	N/A	Y	[77]
Donalisio	The traditional use of Vachellia nilotica for sexually transmitted diseases is substantiated by the antiviral activity of its bark extract against sexually transmitted viruses	2017	In-vitro	Vero cells	Y	V. nilotica chloroform, methanolic and water bark extracts	N	Y	N	Y	ACV resistant HSV-2	Y	[39]
Dong	The Natural Compound Homoharringtonine Presents Broad Antiviral Activity In Vitro and In Vivo	2018	In-vitro, in-vivo	Specific Pathogen-free Chicken Embryo, Vero cells, HEK293Y cells, HeLa cells	Y	Homoharringtonine (HHT)	Y	N	N	N	N/A	Y	[62]
Du	The antiviral activity of arbidol hydrochloride against herpes simplex virus type II (HSV-2) in a mouse model of vaginitis	2019	In-vivo	Vaginitis animals model	Y	Arbidol (ARB)	N	Y	N	N	N/A	Y	[81]
El-Haddad	Brincidofovir (CMX-001) for refractory and resistant CMV and HSV infections in immunocompromised cancer patients: A single-center experience	2016	Clinical trial	4 cancer patients with resistant to CMV or HSV infections	Y	Brincidofovir under emergency IND application	Y	N	N	Y	ACV resistant HSV	Y	[1]
El-Shiekh	Novel Antiviral and Antibacterial Activities of Hibiscus schizopetalus	2020	In-vitro	N/A	Y	70% ethanolic extract (Et-E) of the aerial parts of the Hibiscus schizopetalus (Dyer) Hook.f. (Malvaceae), an ornamental plant	N	N	N	N	N/A	Y	[101]
Eletskaya	Enzymatic synthesis of novel purine nucleosides bearing a chiral benzoxazine fragment	2019	In-vitro	N/A	Y	A series of ribo- and deoxyribonucleosides	Y	N	N	Y	Acyclovir-resistant strain of HSV-1	Y	[76]
Elias	In Vitro Analysis of the Antioxidant and Antiviral Activity of Embelin against Herpes Simplex Virus-1	2021	In-vitro	Vero cells	Y	Embelin	Y	N	N	N	N/A	Y	[122]
Fujimoto	Accumulation of a soluble form of human nectin-2 is required for exerting the resistance against herpes simplex virus type 2 infection in transfected cells	2016	In-vitro	Vero cells	Y	A soluble form of human nectin-2 (hNectin-2Ig), transfected cells expressing the entire ectodomain of nectin-2 fused to the Fc portion of human IgG	N	Y	N	N	N/A	Y	[17]
Fujimoto	Evaluation of the antiviral potential of the soluble forms of glycoprotein D receptors on ocular herpes caused by HSV-1 and HSV-2 infections in a transgenic mouse model	2019	In-vivo	Mice	Y	Transgenic mouse serum containing nectin-1Ig	Y	Y	N	N	N/A	Y	[73]
Fujimoto	Comparison of the antiviral potential among soluble forms of herpes simplex virus type-2 glycoprotein D receptors, herpes virus entry mediator A, nectin-1 and nectin-2, in transgenic mice	2017	In-vitro, in-vivo	Mice	Y	Soluble forms of HVEM, nectin-1 and nectin-2	N	Y	N	N	N/A	Y	[28]
García-Serradilla	Drug repurposing for new, efficient, broad spectrum antivirals	2019	Data Analysis	N/A	Y	Repurposed antiviral drug with different mechanisms of action: digoxin, sunitinib, chloroquine, cyclosporine A and silver nanoparticles in addition to combination therapies with more than one drug	PY	N	N	N	N/A	Y	[65]
Ghaffari	Inhibition of herpes simplex virus type 1 infection by Sambucus ebulus extract in vitro	2021	In-vitro, experimental	Vero cells	Y	Extracts from S. ebulus	Y	N	N	N	N/A	Y	[132]
Ghosh	Ficus religiosa L. bark extracts inhibit infection by herpes simplex virus type 2 in vitro	2016	In-vitro, experimental	Vero cells	Y	F. religiosa extract	N	Y	N	Y	ACV resistant	Y	[20]
González-García	Antimicrobial Activity of Cyclic-Monomeric and Dimeric Derivatives of the Snail-Derived Peptide Cm-p5 against Viral and Multidrug-Resistant Bacterial Strains	2021	In-vitro, experimental	Vero cells, HEK293T cells	Y	Cm-p5 is a snail-derived antimicrobial peptide	N	Y	N	N	N/A	Y	[135]
Greeley	Acyclovir, cidofovir, and amenamevir have additive antiviral effects on herpes simplex virus TYPE 1	2020	In-vitro	Vero cells	Y	(DOE) function in Minitab analyzed the drug-drug interactions of the combination of acyclovir, cidofovir, and amenamevir	Y	N	N	N	N/A	Y	[95]
Hopkins	In Vitro and In Vivo Activity, Tolerability, and Mechanism of Action ofBX795 as an Antiviral against Herpes Simplex Virus 2 Genital Infection	2020	In-vitro, in-vivo	BX795, 8-week-old C57BL/6 female mice	Y	BX795	N	Y	N	N	N/A	Y	[100]
Hou	Antiviral activity of PHA767491 against human herpes simplex virus invitro and in vivo	2017	In-vitro, in-vivo	L929 cells, 8-week-old RIP3 KO mice	Y	More than 1000 compounds for some antiviral drugs were screened by using the model in which HSV-1 directly induced necrosis of L929	Y	Y	N	N	N/A	Y	[37]
Houston	Potentiated virucidal activity of pomegranate rind extract (PRE) and punicalagin against Herpes simplex virus (HSV) when co-administered with zinc (II) ions, and antiviral activity of PRE against HSV and aciclovir-resistant HSV	2017	In-vitro	Vero cells	Y	Pomegranate rind extract (PRE) was used in conjunction with zinc (II) salts	Y	Y	N	Y	ACV-resistant HSV-2	Y	[30]
Huang	Antiviral activity of mitoxantrone dihydrochloride against human herpes simplex virus mediated by suppression of the viral immediate early genes	2019	In-vitro	Mouse fibroblast cells (L929), Vero cells	Y	Mitoxantrone dihydrochloride (MD)	Y	N	N	N	N/A	Y	[86]
Hutterer	Inhibitors of dual-specificity tyrosine phosphorylation-regulated kinases(DYRK) exert a strong anti-herpes viral activity	2017	In-vitro	Human foreskin fibroblasts (HFFs), Vero cells	Y	Novel benzohydrofurane derivatives that target DYRK activity	Y	N	Y	Y	GCV- resistant strain	Y	[38]
Ibáñez	Pharmacological Induction of Heme Oxygenase-1 Impairs Nuclear Accumulation of Herpes Simplex Virus Capsids upon Infection	2017	In-vitro	Vero cells, HeLa cells	Y	Modulating heme oxygenase-1 (HO-1)	N	Y	N	N	N/A	Y	[36]
Ireland	Synthetic α-Hydroxytropolones Inhibit Replication of Wild-Type and Acyclovir-Resistant Herpes Simplex Viruses	2016	In-vitro	Vero cells	Y	Hydroxytropolone pharmacophore	Y	Y	N	Y	(TK)-deficient mutant of HSV-1 and HSV-2	Y	[9]
Ishimaru	MG132 exerts anti-viral activity against HSV-1 by overcoming virus-mediated suppression of the ERK signaling pathway	2020	In-vitro	Vero cells, HepG2, H1299, ME180, MCF7, HeLa cells	Y	Protease inhibitors (TLCK, TPCK, E64, bortezomib, or MG132)	Y	N	N	N	N/A	Y	[99]
Jaishankar	An off-target effect of BX795 blocks herpes simplex virus type 1 infection of the eye	2018	In-vitro, in-vivo, ex-vivo	Human corneal epithelial (HCE) cells, Mouse model, porcine and human cornea organ culture	Y	BX795 and its potential synergism with trifluridine (TFT)	Y	N	N	N	N/A	Y	[56]
Jin	Pentagalloylglucose Blocks the Nuclear Transport and the Process of Nucleocapsid Egress to Inhibit HSV-1 Infection	2015	In-vitro	Vero cells	Y	Pentagalloylglucose (PGG)-induced inhibition of nuclear transport and nucleocapsid egress	Y	N	N	Y	A TK mutant from HSV-1 and two ACV-resistant clinical HSV-1 strains	Y	[6]
Jones	Modified cyclodextrins as broad-spectrum antivirals	2020	In-vitro	Vero cells	Y	Cyclodextrins modified with mercaptoundecane sulfonic acids	Y	Y	N	Y	ACV resistant HSV-2	Y	[93]
Kalke	Herpes Simplex Virus Type 1 Clinical Isolates Respond to UL29-TargetedsiRNA Swarm Treatment Independent of Their Acyclovir Sensitivity	2020	In-vitro	Vero cells	Y	Enzymatically synthesized siRNA swarms	Y	N	N	Y	ACV resistant HSV-1	Y	[98]
Kannan	Anti-herpes virus activity of the carnivorous botanical, Sarracenia purpurea	2020	In-vitro	Vero cells	Y	S. purpurea extract	Y	N	N	N	N/A	Y	[87]
Karpov	[A Plasmid-Expressed CRISPR/Cas9 System Suppresses Replication of HSV Type I in a Vero Cell Culture]	2019	In-vitro	Vero cells	Y	Genome editing via prokaryotic plasmid CRISPR/Cas9	Y	N	N	N	N/A	Y	[79]
Katsumata	Antiviral efficacy of the helicase-primase inhibitor amenamevir in murinemodels of severe herpesvirus infection	2018	In-vivo	Mice	Y	Amenamevir, a helicase-primase inhibitor	Y	N	Y	N	N/A	Y	[43]
Kaushik	Antiviral potential and mode of action of Indigofera heterantha against HSV-2 by targeting the early stages of infection	2016	In-vitro, in-vivo	Mice and plaque reduction assays	Y	Extract of roots of the plant Indigofera heterantha	N	Y	N	N	N/A	Y	[18]
Kim	Quercus acuta Thunb. (Fagaceae) and Its Component, Isoquercitrin, InhibitHSV-1 Replication by Suppressing Virus-Induced ROS Production and NF-κB Activation	2021	In-vitro	Vero cells	Y	Quercus acuta Thunb (Fagaceae) (QA) extract	Y	N	N	N	N/A	Y	[127]
Kim	Mori ramulus and its Major Component Morusin Inhibit Herpes Simplex Virus Type 1 Replication and the Virus-Induced Reactive Oxygen Species	2020	In-vitro	Vero cells	Y	Mori ramulus (the young twig of Morus alba L.)	Y	N	N	N	N/A	Y	[103]
Kongyingyoes	3,19-isopropylideneandrographolide suppresses early gene expression of drug-resistant and wild type herpes simplex viruses	2016	In-vitro	Vero cells	Y	A diterpenoid lactone, 3,19-isopropylideneandrographolide (IPAD) compound isolated from Andrographis	Y	Y	N	Y	ACV-resistant and (TK) deficient	Y	[14]
Kumar	Inhibition of herpes simplex virus-1 infection by MBZM-N-IBT: in silico and in vitro studies	2021	In-vitro	Vero cells	Y	MBZM-N-IBT impact against HSV-1	Y	N	N	N	N/A	Y	[133]
Labrunie	UL23, UL30, and UL5 characterization of HSV1 clinical strains isolated from hematology department patients	2019	In-vitro	N/A	N	Genetic variants	N	N	N	N	N/A	N	[84]
Le-Trilling	Broad and potent antiviral activity of the NAE inhibitor MLN4924	2016	In-vitro	N/A	Y	NAE inhibitor MLN4924	Y	Y	N	Y	ACV, CDV and PFA resistant HSV-1	Y	[19]
Lebrun	Varicella-Zoster Virus ORF9p Binding to Cellular Adaptor Protein Complex 1Is Important for Viral Infectivity	2018	In-vitro	Yeast cells	N	ORF9p proteins	N	N	N	N	N/A	N	[139]
Lee	Efficacy of brincidofovir as prophylaxis against HSV and VZV in hematopoietic cell transplant recipients	2018	In-vivo	2710 patient-days	Y	Brincidofovir a lipid conjugate of cidofovir	Y	Y	Y	N	N/A	Y	[48]
Lei	Preparation of a monoPEGylated derivative of cyanovirin-N and its virucidal effect on acyclovir-resistant strains of herpes simplex virus type 1	2019	In-vivo	N/A	Y	Cyanovirin-N (CV-N) more specifically LCV-N as the most potent of three compounds	Y	N	N	Y	ACV resistant	Y	[2]
Li	Amentoflavone Inhibits HSV-1 and ACV-Resistant Strain Infection by Suppressing Viral Early Infection	2019	In-vivo	N/A	Y	Amentoflavone, a naturally occurring biflavonoid	Y	N	N	Y	ACV resistant	Y	[67]
Li	Anti-herpes simplex virus type 1 activity of Houttuynoid A, a flavonoidfrom Houttuynia cordata Thunb	2017	In-vitro, in-vivo	Mice	Y	Houttuynia A cordata Thunb. water extract, a new type of flavonoid isolated from H. cordata	Y	Y	Y	N	N/A	Y	[25]
Liu	Antiviral activities of Janus-type nucleosides and their related oxime-intermediates	2018	In-vitro	Vero cells	Y	Janus-type nucleosides combining the natural genetic alphabets into a singular nucleoside structural unit	Y	N	N	N	N/A	Y	[41]
Liu	Harringtonine Inhibits Herpes Simplex Virus Type 1 Infection by Reducing Herpes Virus Entry Mediator Expression	2021	In-vitro	Vero cells	Y	Harringtonine	Y	N	N	Y	(TK) mutation in HSV-1	Y	[116]
Lopes	Sulfonated and Carboxymethylated β-Glucan Derivatives with Inhibitory Activity against Herpes and Dengue Viruses	2021	In-vitro	Vero cells	Y	(1→3)(1→6)-β-D-glucan, botryosphaeran, similar to an anionic polysaccharide	Y	N	N	Y	ACV resistant	Y	[128]
Luganini	Effective deploying of a novel DHODH inhibitor against herpes simplex type1 and type 2 replication	2021	In-vitro	Vero cells	Y	MEDS433 a pyrimidine synth inhibitor	Y	Y	N	N	N/A	Y	[129]
Ma	Herpes simplex virus type 1 (HSV-1) specific T-cell generation fromHLA-A1- and HLA-A2-positive donors for adoptive immunotherapy	2016	In-vitro	Peripheral blood mononuclear cells from HLA-A1 and HLA-A2 HSV-seropositive hereditary hemochromatosis donors	Y	HSV-1-specific T cells	Y	N	N	N	N/A	Y	[12]
Ma	Assessment of a new arbidol derivative against herpes simplex virus II inhuman cervical epithelial cells and in BALB/c mice	2019	In-vitro	HCE cells	Y	Arbidol derivative (ARD)	N	Y	N	N	N/A	Y	[75]
Maizel	Study of the Extremely-Tolerant Brevibacterium linens AE038-8 with Antiviral Activity Against Herpes Simplex Virus Type 1	2021	In-vitro	N/A	Y	B. linens AE038-8	Y	N	N	Y	ACV resistant	Y	[113]
Mandalari	Simulated human digestion of N1-aryl-2-arylthioacetamidobenzimidazoles and their activity against Herpes-simplex virus 1 in vitro	2019	In-vitro	N/A	Y	NAAB-496 and NAAB-503	Y	N	N	N	N/A	Y	[72]
Marcocci	The Amphibian Antimicrobial Peptide Temporin B Inhibits In Vitro Herpes Simplex Virus 1 Infection	2018	In-vitro	Vero cells, human epithelial cells	Y	Temporin B (TB)	Y	N	N	N	N/A	Y	[54]
Marino-Merlo	Anti-herpes simplex virus 1 and immunomodulatory activities of a poly-γ-glutamic acid from Bacillus horneckiae strain APA of shallow vent origin	2017	In-vitro	HEp-2 cells, U937 cells,	Y	Poly-γ-glutamic acid (γ-PGA-APA)	Y	N	N	N	N/A	Y	[29]
Mello	Perillyl alcohol and perillic acid exert efficient action upon HSV-1maturation and release of infective virus	2020	In-vitro	Vero cells	Y	Monoterpenes perillyl alcohol (POH) and perillic acid (PA)	Y	N	N	N	N/A	Y	[92]
Mishra	Herbal Gel Formulation Developed for Anti-Human Immunodeficiency Virus(HIV)-1 Activity Also Inhibits In Vitro HSV-2 Infection	2018	In-vitro	Vero cells	Y	Polyherbal gel formulation (aqueous gel formulation comprising of 50% ethanolic extracts prepared from stem bark of Acacia catechu, leaves of Lagerstroemia speciosa, and fruits of Terminalia chebula & Phyllanthus emblica)	N	Y	N	N	N/A	Y	[59]
Mohammed	Synthesis and anti-HSV activity of tricyclic penciclovir and hydroxybutyl guanine derivatives	2019	In-vitro	Human embryonic lung (HEL) cell, Vero cells, HeLa cells, MDCK cells	Y	Novel tricyclic derivatives	Y	Y	N	Y	ACV resistant, (TK-)	Y	[69]
Monjo	Photodynamic Inactivation of Herpes Simplex Viruses	2018	In-vitro	HeLa, HEK293A, Vero cells	Y	Orthoquin in sub-cytotoxic doses	Y	Y	N	N	N/A	Y	[55]
Moshaverinia	Evaluation of the effect of hydro alcoholic extract of cinnamon on herpes simplex virus-1	2020	In-vitro	N/A	Y	Hydroalcoholic extract of cinnamon	Y	N	N	N	N/A	Y	[94]
Musarra-Pizzo	The Antimicrobial and Antiviral Activity of Polyphenols from Almond(Prunus dulcis L.) Skin	2019	In-vitro	Vero cells	Y	Natural almond skin (NS MIX)	Y	N	N	N	N/A	Y	[68]
Novoa	Antiviral Activity of Myticin C Peptide from Mussel: an Ancient Defense against Herpesviruses	2016	In-vitro	Vero cells	Y	Myticin C Peptide	Y	Y	N	N	N/A	Y	[8]
Paavilainen	Topical treatment of herpes simplex virus infection with enzymatically created siRNA swarm	2017	In-vivo	BALB/c mice	Y	Treated with a swarm of enzymatically created, Dicer-substrate small interfering RNA (siRNA) molecules that targeted the HSV gene UL29	Y	N	N	N	N/A	Y	[22]
Parsania	Antiviral screening of four plant extracts against acyclovir resistant herpes simplex virus type-1	2017	In-vitro	N/A	Y	Methanolic extract of four plants	Y	N	N	N	N/A	Y	[24]
PiresdeMello	Aminomethylnaphthoquinones and HSV-1: in vitro and in silico evaluations of potential antivirals	2016	In-vitro	Vero cells	Y	Three 2-aminomethyl-3-hydroxy-1,4-naphthoquinones	Y	N	N	N	N/A	Y	[3]
Pradhan	Herpes simplex virus virucidal activity of MST-312 and epigallocatechin gallate	2018	In-vitro	N/A	Y	MST-312	Y	N	N	N/A	N/A	Y	[63]
Praena	Amidic derivatives of valproic acid, valpromide and valnoctamide, inhibitHSV-1 infection in oligodendrocytes	2019	In-vivo	Glial cells	Y	Two amidic derivatives of valproic acid (VPA) - valpromide (VPD) and valnoctamide (VCD)	Y	N	N	N	N/A	Y	[78]
Pujol	Polyhydroxylated sulfated steroids derived from 5α-cholestanes as antiviral agents against herpes simplex virus	2016	In-vitro	Human cells lines, vero cells	Y	Twelve polyhydroxylated sulfated steroids synthesized from a 5α-cholestane skeleton with different substitutions in C-2, C-3 and C-6	Y	Y	N	N	N/A	Y	[16]
Quenelle	Efficacy of pritelivir and acyclovir in the treatment of herpes simplex virus infections in a mouse model of herpes simplex encephalitis	2017	In-vitro	Mice	Y	Pritelivir, a helicase-primase inhibitor, has excellent in vitro and in vivo activity against human herpes simplex virus (HSV). Mice lethally infected with HSV type 1 or 2, including acyclovir-resistant strains, were treated 72 h after infection for 7 days with pritelivir or acyclovir.	Y	Y	N	Y	ACV resistant	Y	[35]
Rechenchoski	Mangiferin: A promising natural xanthone from Mangifera indica for the control of acyclovir - resistant herpes simplex virus 1 infection	2020	In-vitro, in-vivo	Vero cells	Y	M. Indica (Mangiferin; a mango extract)	Y	N	N	Y	ACV-resistant HSV-1	Y	[106]
Rittà	Antiviral Activity of a Arisaema Tortuosum Leaf Extract and Some of its Constituents against Herpes Simplex Virus Type 2	2020	In-vitro	Vero cells	Y	Arisaema tortuosum, a plant medicine from India	Y	Y	N	Y	Acyclovir-resistant HSV-2	Y	[91]
Ruzsics	A Novel, Broad-Acting Peptide Inhibitor of Double-Stranded DNA Virus Gene Expression and Replication	2020	In-vitro	Vero cells	Y	A novel peptide called TAT-I24	Y	N	N	Y	ACV resistant	Y	[90]
Sacchelli	Botryosphaeran and sulfonated derivatives as novel antiviral agents for herpes simplex and dengue fever	2019	In-vitro	Vero cells	Y	Botryosphaeran, a fungal exocellular (1 → 3)(1 → 6)-β-D glucan devoid of sulfate groups	Y	N	N	N	N/A	Y	[74]
SadeghEhdaei	Cellular miR-101-1 Reduces Efficiently the Replication of HSV-1 in HeLa Cells	2021	In-vitro	HeLa cells	Y	Hsa-miR-101-1	Y	N	N	N	N/A	Y	[114]
Sanchez	Development and evaluation of a host-targeted antiviral that abrogates herpes simplex virus replication through modulation of arginine-associated metabolic pathways	2016	In-vitro	Primary human corneal epithelial cells (HCEC)	Y	A pegylated recombinant human Arginase I (peg-ArgI)	Y	Y	N	Y	Polymerase (PAAr5) or thymidine kinase (tkLTRZ1; tkG7dG.2) genes	Y	[11]
Sasaki	In vitro and in vivo antiherpetic effects of(1R,2R)-1-(5'-methylful-3'-yl)propane-1,2,3-triol	2016	In-vitro, in-vivo	Female BALB/c mice 5–6 weeks old	Y	MFPT	Y	Y	N	Y	ACV resistant HSV-1	Y	[15]
Schneider	Early Steps in Herpes Simplex Virus Infection Blocked by a Proteasome Inhibitor	2019	In-vitro	Vero cells, human foreskin fibroblasts	Y	Bortezomib and many of its property against HSV	Y	Y	N	Y	ACV resistant	Y	[83]
Shabani	Inhibition of herpes simplex virus type 1 replication by novelhsa-miR-7704 in vitro	2019	In-vitro	HeLa cells	Y	A novel miRNA (hsa-miR-7704), expressed in macrophages	Y	N	N	N	N/A	Y	[71]
Shan	Viral UL8 Is Involved in the Antiviral Activity of Oleanolic Acid AgainstHSV-1 Infection	2021	In-vitro	Vero cells, Human immortalized keratinocyte cell line (HaCaT)	Y	Oleanolic acid, a pentacyclic triterpenoid widely existing in natural product	Y	N	N	Y	TK mutant from HSV-1 and two clinical ACV-resistant HSV-1 strains	Y	[130]
Shao	Poly(dA:dT) Suppresses HSV-2 Infection of Human Cervical Epithelial Cells Through RIG-I Activation	2021	In-vitro	Human endocervical epithelia (End1) cells	Y	Poly (dA:dT) treatment of End1/E6E7 cells	N	Y	N	N	N/A	Y	[124]
Sharifi-Rad	Susceptibility of herpes simplex virus type 1 to monoterpenes thymol, carvacrol, p-cymene and essential oils of Sinapis arvensis L., Lallemantia royleana Benth. and Pulicaria vulgaris Gaertn	2017	In-vitro	Vero cells	Y	Three monoterpenes (thymol, carvacrol and p-cymene) and three essential oils	Y	N	N	N	N/A	Y	[34]
Sharifi-Rad	Antiviral activity of Veronica persica Poir. on herpes virus infection	2018	In-vitro	Vero cells	Y	Veronica persica Poir extract	Y	Y	N	N	N/A	Y	[51]
Shiraki	Helicase-primase inhibitor amenamevir for herpesvirus infection: Towards practical application for treating herpes zoster	2017	N/A	N/A	Y	Helicase-primase inhibitors (HPIs) inhibit the progression of the replication fork ( initial step in DNA synthesis to separate the double strand into two single strands). The HPIs amenamevir and pritelivir have a novel mechanism of action, once-daily administration with nonrenal excretory characteristics, and clinical efficacy for genital herpes.	Y	N	Y	N	N/A	PY	[21]
Shiraki	Amenamevir, a Helicase-Primase Inhibitor, for the Optimal Treatment of Herpes Zoster	2021	N/A	N/A	Y	Amenamevir and synergism with acyclovir.	Y	N	Y	Y	Amenamevir-resistant viruses with changes in the helicase and primase of amenamevir-resistant HSV mutants	Y	[121]
Spengler	Antiviral, Antimicrobial and Antibiofilm Activity of Selenoesters and Selenoanhydrides	2019	In-vitro	Vero cells	Y	Selenoesters and selenium isostere	N	Y	N	N	N/A	Y	[66]
Stegman	Volatile Acid-Solvent Evaporation (VASE): Molecularly Homogeneous Distribution of Acyclovir in a Bioerodable Polymer Matrix for Long-Term Treatment of Herpes Simplex Virus-1 Infections	2018	In-vitro	Vero cells	Y	Bioerodable polymer polycaprolactone	Y	N	N	N	N/A	Y	[40]
Suryawanshi	Bacterial Pigment Prodigiosin Demonstrates a Unique Antiherpes virus Activity That Is Mediated through Inhibition of Prosurvival Signal Transducers	2020	In-vitro, ex-vivo, in-vivo	Human corneal epithelial (HCE) cells, HeLa cells, C57BL/6 mice, porcine corneal model, whole pig eyes	Y	Prodigiosin (PG)	Y	Y	N	N	N/A	Y	[109]
Tavakoli	Inhibition of herpes simplex virus type 1 by copper oxide nanoparticles	2019	In-vitro	Vero cells	Y	Copper oxide nanoparticles (CuO-NPs) on HSV-1 infection	Y	N	N	N	N/A	Y	[70]
Tintori	Rhodanine derivatives as potent anti-HIV and anti-HSV microbicides	2018	In-vitro	Vero cells, human CD4+ lymphocytes	Y	Rhodanine derivatives	Y	Y	N	Y	ACV resistant HSV-2	Y	[61]
Toscani	Synthesis and Biological Evaluation of Amidinourea Derivatives against Herpes Simplex Viruses	2021	In-vitro	Vero cells	Y	Amidinourea analogues of moroxydine	Y	Y	N	N	N/A	Y	[118]
Toulabi	The efficacy of olive leaf extract on healing herpes simplex virus labialis: A randomized double-blind study	2021	Randomized double-blind clinical trial	66 human patients diagnosed with HSV-1	Y	Comparison of 2% OLE cream or 5% acyclovir cream five times a day for six days	Y	N	N	N	N/A	Y	[119]
Tyo	pH-responsive delivery of Griffithsin from electrospun fibers	2019	In-vitro	Vaginal keratinocyte, endocervical, and ectocervical cells, TZM-bl cell	Y	H-responsive fibers comprised of poly(lactic-co-glycolic acid) (PLGA) or methoxypolyethylene glycol-b-PLGA (mPEG-PLGA) with varying ratios of poly(n-butyl acrylate-co-acrylic acid) (PBA-co-PAA), to selectively release griffithsin (GRFT) under pH-conditions that mimic semen introduction	N	N	N	N	N/A	Y	[80]
Uhlig	Helicase primase inhibitors (HPIs) are efficacious for therapy of human herpes simplex virus (HSV) disease in an infection mouse model	2021	In-vitro, in-vivo	Female BALB/c mice (8 weeks old), Vero cells	Y	Diverse racemates of the sulfonimidoyl thiazole amide class compounds	Y	Y	N	Y	ACV-resistant HSV-1 and HSV-2	Y	[137]
Urbancikova	Efficacy of Pleuran (β-Glucan from Pleurotus ostreatus) in the Managementof Herpes Simplex Virus Type 1 Infection	2020	Clinical trial	90 human patients over 6years with herpes simplex facialis/labialis	Y	β-glucanpleuran (insolubleβ-1,3/1,6-D-glucan isolated from Pleurotus ostreatus) based supplements	Y	N	N	N	N/A	Y	[88]
Vanheule	Basic chemokine-derived glycosaminoglycan binding peptides exert antiviral properties against dengue virus serotype 2, herpes simplex virus-1 and respiratory syncytial virus	2015	In-vitro	Chinese Hamster ovary, human embryonic lung and human cervical carcinoma (HeLa) cells	Y	COOH-terminal peptides of CXCL9 and CXCL12γ for their affinity to GAGs and KD values	Y	Y	N	N	N/A	Y	[7]
Viegas	Antiviral activity of 1,4-disubstituted-1,2,3-triazoles against HSV-1 invitro and effects of amino acid changes in drug-resistant α and βherpesviruses DNA polymerase	2020	In-vitro	Human fibroblast cells	Y	Triazole compounds	Y	N	N	Y	ACV resistant HSV-1	Y	[96]
VilasBoas	Linear antimicrobial peptides with activity against herpes simplex virus 1and Aichi virus	2017	In-vitro	N/A	Y	Various antimicrobial peptides	Y	N	N	N	N/A	Y	[23]
Vilhelmova-Ilieva	Antiviral Activity of Rosa damascena Mill. and Rosa alba L. Essential Oils against the Multiplication of Herpes Simplex Virus Type 1 Strains Sensitive and Resistant to Acyclovir	2021	In-vitro	Madin-Darby bovine kidney (MDBK) cells	Y	Rosa damascena Mill. and Rosa alba L. essential oils	Y	N	N	Y	ACV resistant	N	[131]
Wang	Guanidine modifications enhance the anti-herpes simplex virus activity of(E,E)-4,6-bis(styryl)-pyrimidine derivatives in vitro and in vivo	2020	In-vitro, in-vivo	Vero cells	Y	Guanidine-modified (E,E)-4,6-bis(styryl)-pyrimidine (BS-pyrimidine) derivative compound 5d	Y	Y	N	N	N/A	Y	[108]
Wang	Anti-HSV-1 activity of Aspergilli peptide D, a cyclic pentapepetide isolated from fungus Aspergillus sp. SCSIO 41501	2020	In-vitro	N/A	Y	Aspergillipeptide D	Y	N	N	N	N/A	N	[107]
Whitley	Clinical management of herpes simplex virus infections: past, present, and future	2018	N/A	N/A	N	N/A	N	N	N	N	N/A	N/A	[57]
Wright	Inhibition of Herpes Simplex Viruses, Types 1 and 2, by Ginsenoside20(S)-Rg3	2020	In-vitro	Vero cells	Y	Ginsenosides derived from Panax ginseng	Y	Y	N	N	N/A	Y	[105]
Ye	Lupeol impairs herpes simplex virus type 1 replication by inhibiting the promoter activity of the viral immediate early gene α0	2021	In-vitro	N/A	Y	Lupeol, a triterpenoid compound	Y	N	N	Y	ACV resistant	Y	[115]
Zhang	NSC23766 and Ehop016 Suppress Herpes Simplex Virus-1 Replication by Inhibiting Rac1 Activity	2021	In-vitro	Vero cells	Y	Ras-related C3 botulinum toxin substrate 1 Rac1 as a target using Rac1-specific inhibitors, titled NSC23766 and Ehop016	Y	N	N	N	N/A	Y	[117]
Zhou	Anti-HSV-1 effect of dihydromyricetin from Ampelopsis grossedentata via the TLR9-dependent anti-inflammatory pathway	2020	In-vitro, experimental study	Vero cells	Y	A flavonoid compound dihydromyricetin (DHM) from Ampelopsis grossedentata	Y	N	N	N	N/A	Y	[97]
Zígolo	Chemoenzymatic synthesis of new derivatives of glycyrrhetinic acid with antiviral activity. Molecular docking study	2018	In-vitro, experimental study	Vero cells	Y	Synthesized GA derivative, 4d (N-(3-acetylglycyrrhetinoyl)-2-amino-1-propanol)	Y	N	N	Y	ACV resistant HSV-1	Y	[42]
Zinser	A new promising candidate to overcome drug resistant herpes simplex virus infections	2017	In-vitro, experimental study	Vero cells	Y	Synthesized SC95377	Y	Y	N	Y	ACV and multi-resistant resistant HSV-1 and HSV-2	Y	[33]

Assessment of Risk of Bias

Quality assessment using the Cochrane Risk of Bias Tool for randomized trials is shown in Table 2. All studies did not report sufficient information to assess other sources of bias, so this area of judgement was excluded from Table 2. Within the domains assessed in the Cochrane Risk of Bias Tool, the ‘Blinding of Outcome Assessment” domain had the greatest number of studies (25) rated as “high risk” of bias (Table 2).

**Table 2:** Cochrane risk of bias assessment

Cochrane Risk of Bias Assessment							
Author	Title	Random Sequence Generation	Allocation Concealment	Blinding of Outcome Assessment	Incomplete Outcome Data	Selective Reporting	Citation
Agostinho	Cucumis melo pectin as potential candidate to control herpes simplex virus infection	Low	Unclear	Unclear	Low	Unclear	[123]
Al-Salahi	Molecular docking study and antiviral evaluation of2-thioxo-benzo[g]quinazolin-4(3H)-one derivatives	Low	Unclear	Unclear	Low	Unclear	[10]
Alvarez	Cetylpyridinium chloride blocks herpes simplex virus replication in gingival fibroblasts	Low	Unclear	Unclear	Low	Unclear	[104]
Andrei	The Anti-Human Immunodeficiency Virus Drug Tenofovir, a Reverse Transcriptase Inhibitor, Also Targets the Herpes Simplex Virus DNA Polymerase	Low	Unclear	Unclear	Low	Unclear	[49]
Andronova	Study of Antiherpetic Efficiency of Phosphite of Acycloguanosine Able to Overcome the Barrier of Resistance to Acyclovir	Unclear	Unclear	Unclear	Unclear	Unclear	[13]
Arunkumar	Study on antiviral activities, drug-likeness and molecular docking of bioactive compounds of Punica granatum L. to Herpes simplex virus - 2(HSV-2)	Low	Unclear	Unclear	Low	Unclear	[52]
Awad	Synthesis and Evaluation of Some Uracil Nucleosides as Promising Anti-Herpes Simplex Virus 1 Agents	Low	Unclear	Unclear	Low	Unclear	[111]
Barboza	In vitro effects of bufotenine against RNA and DNA viruses	Low	Unclear	Unclear	Low	Unclear	[126]
Bauer	Antibody-based immunotherapy of aciclovir resistant ocular herpes simplex virus infections	Unclear	Unclear	Unclear	Unclear	Unclear	[27]
Ben-Amor	Phytochemical Characterization of Olea europea Leaf Extracts and Assessment of Their Anti-Microbial and Anti-HSV-1 Activity	Low	Unclear	Unclear	Low	Unclear	[125]
Benassi-Zanqueta	Evaluation of anti-HSV-1 activity and toxicity of hydroethanolic extract of Tanacetum parthenium (L.) Sch.Bip. (Asteraceae)	Low	Unclear	Unclear	Low	Unclear	[64]
Benzekri	Anti HSV-2 activity of Peganum harmala (L.) and isolation of the active compound	Low	Unclear	Unclear	Low	Unclear	[32]
Bereczki	Synthesis of Antiviral Perfluoroalkyl Derivatives of Teicoplanin and Vancomycin	Low	Unclear	Unclear	Low	Unclear	[89]
Bhutta	Peptide Inhibitor of Complement C1, RLS-0071, Reduces Zosteriform Spread of Herpes Simplex Virus Type 1 Skin Infection and Promotes Survival in Infected Mice	Unclear	Low	Unclear	Low	Unclear	[134]
Bhutta	Ginkgolic Acid Inhibits Herpes Simplex Virus Type 1 Skin Infection and Prevents Zosteriform Spread in Mice	Unclear	Low	Unclear	Low	Unclear	[120]
Bisignano	Almond Skin Extracts Abrogate HSV-1 Replication by Blocking Virus Binding to the Cell	Low	Unclear	Unclear	Low	Unclear	[26]
Bonvicini	Hemidesmus indicus (L.) R. Br. extract inhibits the early step of herpes simplex type 1 and type 2 replication	Low	Unclear	Unclear	Low	Unclear	[44]
Brenner	The Molecular Tweezer CLR01 Inhibits Antibody-Resistant Cell-to-Cell Spread of Human Cytomegalovirus	Low	Low	Low	Low	Unclear	[136]
Brezáni	Anti-Infectivity against Herpes Simplex Virus and Selected Microbes and Anti-Inflammatory Activities of Compounds Isolated from Eucalyptusglobulus Labill	Low	Unclear	Unclear	Low	Unclear	[138]
Cagno	In vitro anti-herpes simplex virus-2 activity of Salvia desoleana Atzei &V. Picci essential oil	Low	Unclear	Unclear	Low	Unclear	[31]
Castillo	Anti-herpetic Activity of Macrocystis pyrifera and Durvillaea antarctica Algae Extracts Against HSV-1 and HSV-2	Low	Low	High	Low	Unclear	[110]
Chen	Targeting Aryl Hydrocarbon Receptor Signaling Enhances Type I Interferon-Independent Resistance to Herpes Simplex Virus	Low	Unclear	Unclear	Low	Unclear	[112]
Crameri	MxB is an interferon-induced restriction factor of human herpes viruses	Low	Low	Low	Low	Unclear	[53]
Criscuolo	Synergy evaluation of anti-Herpes Simplex Virus type 1 and 2 compounds acting on different steps of virus life cycle	Low	Low	Unclear	HIgh	Low	[46]
Čulenová	Multiple In vitro biological effects of phenolic compounds from Morus albaroot bark	Low	Low	Unclear	Unclear	Unclear	[85]
D'Aiuto	R430: A potent inhibitor of DNA and RNA viruses	Low	Unclear	Unclear	Low	Unclear	[47]
Dai	Antiviral Effect of Retro-2.1 against Herpes Simplex Virus Type 2 In Vitro	Low	Unclear	Unclear	Low	Unclear	[50]
Dai	Antiviral Effects of ABMA against Herpes Simplex Virus Type 2 In Vitro and In Vivo	Low	Unclear	Unclear	Unclear	Unclear	[58]
Deback	Antiviral effects of Cacicol(®), a heparan sulfate biomimetic for corneal regeneration therapy, for herpes simplex virus type-1 and varicella zoster virus infection	Low	Unclear	Unclear	Low	Unclear	[60]
Derby	Griffithsin carrageenan fast dissolving inserts prevent SHIV HSV-2 and HPV infections in vivo	Low	Low	Unclear	Unclear	Unclear	[45]
Deschamps	Discovery of Small-Molecule Inhibitors Targeting the E3 Ubiquitin Ligase Activity of the Herpes Simplex Virus 1 ICP0 Protein Using an In Vitro High-Throughput Screening Assay	Unclear	Low	Low	Unclear	Unclear	[82]
Ding	T-type calcium channels blockers inhibit HSV-2 infection at the late stage of genome replication	Unclear	Low	Unclear	Low	Unclear	[102]
Ding	Cellular Signaling Analysis shows antiviral, ribavirin-mediated ribosomal signaling modulation	Low	High	Unclear	Low	Unclear	[77]
Donalisio	The traditional use of Vachellia nilotica for sexually transmitted diseases is substantiated by the antiviral activity of its bark extract against sexually transmitted viruses	Low	Unclear	N/A	Low	Unclear	[39]
Dong	The Natural Compound Homoharringtonine Presents Broad Antiviral Activity In Vitro and In Vivo	Unclear	Low	N/A	Low	Low	[62]
Du	The antiviral activity of arbidol hydrochloride against herpes simplex virus type II (HSV-2) in a mouse model of vaginitis	Unclear	Low	Low	Unclear	Unclear	[81]
El-Haddad	Brincidofovir (CMX-001) for refractory and resistant CMV and HSV infections in immunocompromised cancer patients: A single-center experience	Unclear	Low	Unclear	N/A	Low	[1]
El-Shiekh	Novel Antiviral and Antibacterial Activities of Hibiscus schizopetalus	Low	Unclear	Unclear	N/A	Unclear	[101]
Eletskaya	Enzymatic synthesis of novel purine nucleosides bearing a chiral benzoxazine fragment	Unclear	High	Low	Unclear	N/A	[76]
Elias	In Vitro Analysis of the Antioxidant and Antiviral Activity of Embelin against Herpes Simplex Virus-1	Low	Unclear	Unclear	Low	High	[122]
Fujimoto	Accumulation of a soluble form of human nectin-2 is required for exerting the resistance against herpes simplex virus type 2 infection in transfected cells	Unclear	Unclear	Unclear	Low	N/A	[17]
Fujimoto	Evaluation of the antiviral potential of the soluble forms of glycoprotein D receptors on ocular herpes caused by HSV-1 and HSV-2 infections in a transgenic mouse model	Low	Low	High	N/A	Unclear	[73]
Fujimoto	Comparison of the antiviral potential among soluble forms of herpes simplex virus type-2 glycoprotein D receptors, herpes virus entry mediator A, nectin-1 and nectin-2, in transgenic mice	Low	Low	High	Unclear	N/A	[28]
García-Serradilla	Drug repurposing for new, efficient, broad-spectrum antivirals	Low	Unclear	High	Unclear	N/A	[65]
Ghaffari	Inhibition of herpes simplex virus type 1 infection by Sambucus ebulus extract in vitro	Low	Unclear	Low	Unclear	Unclear	[132]
Ghosh	Ficus religiosa L. bark extracts inhibit infection by herpes simplex virus type 2 in vitro	Low	Unclear	Unclear	Low	Low	[20]
González-García	Antimicrobial Activity of Cyclic-Monomeric and Dimeric Derivatives of the Snail-Derived Peptide Cm-p5 against Viral and Multidrug-Resistant Bacterial Strains	Unclear	Low	Unclear	Unclear	Low	[135]
Greeley	Acyclovir, cidofovir, and amenamevir have additive antiviral effects on herpes simplex virus TYPE 1	Unclear	Low	Low	Unclear	Unclear	[95]
Hopkins	In Vitro and In Vivo Activity, Tolerability, and Mechanism of Action of BX795 as an Antiviral against Herpes Simplex Virus 2 Genital Infection	Unclear	Low	Unclear	Unclear	Low	[100]
Hou	Antiviral activity of PHA767491 against human herpes simplex virus in vitro and in vivo	Low	N/A	Unclear	Low	Low	[37]
Houston	Potentiated virucidal activity of pomegranate rind extract (PRE) and punicalagin against Herpes simplex virus (HSV) when co-administered with zinc (II) ions, and antiviral activity of PRE against HSV and acyclovir-resistant HSV	Unclear	Low	Low	Unclear	Unclear	[30]
Huang	Antiviral activity of mitoxantrone dihydrochloride against human herpes simplex virus mediated by suppression of the viral immediate early genes	Low	Low	Unclear	N/A	Low	[86]
Hutterer	Inhibitors of dual-specificity tyrosine phosphorylation-regulated kinases (DYRK) exert a strong anti-herpes viral activity	Unclear	Low	Low	Unclear	Low	[38]
Ibáñez	Pharmacological Induction of Heme Oxygenase-1 Impairs Nuclear Accumulation of Herpes Simplex Virus Capsids upon Infection	N/A	Low	High	Unclear	Low	[36]
Ireland	Synthetic α-Hydroxytropolones Inhibit Replication of Wild-Type and Acyclovir-Resistant Herpes Simplex Viruses	Unclear	Low	Unclear	Low	Unclear	[9]
Ishimaru	MG132 exerts anti-viral activity against HSV-1 by overcoming virus-mediated suppression of the ERK signaling pathway	Low	Low	N/A	Unclear	Low	[99]
Jaishankar	An off-target effect of BX795 blocks herpes simplex virus type 1 infection of the eye	Low	N/A	Unclear	Low	Unclear	[56]
Jin	Pentagalloyl glucose Blocks the Nuclear Transport and the Process of Nucleocapsid Egress to Inhibit HSV-1 Infection	Low	Unclear	Unclear	Low	N/A	[6]
Jones	Modified cyclodextrins as broad-spectrum antivirals	Low	Low	Unclear	Unclear	Unclear	[93]
Kalke	Herpes Simplex Virus Type 1 Clinical Isolates Respond to UL29-Targeted siRNA Swarm Treatment Independent of Their Acyclovir Sensitivity	Low	Low	Unclear	Low	Unclear	[98]
Kannan	Anti-herpes virus activity of the carnivorous botanical, Sarraceniapurpurea	N/A	Low	Low	Unclear	N/A	[87]
Karpov	[A Plasmid-Expressed CRISPR/Cas9 System Suppresses Replication of HSV Type I in a Vero Cell Culture]	High	Low	Unclear	Unclear	Low	[79]
Katsumata	Antiviral efficacy of the helicase-primase inhibitor amenamevir in murine models of severe herpesvirus infection	Low	Unclear	Unclear	Unclear	Low	[43]
Kaushik	Antiviral potential and mode of action of Indigofera heterantha against HSV-2 by targeting the early stages of infection	Low	Unclear	Unclear	Unclear	Low	[18]
Kim	Quercus acuta Thunb. (Fagaceae) and Its Component, Isoquercitrin, InhibitHSV-1 Replication by Suppressing Virus-Induced ROS Production and NF-κB Activation	Low	Unclear	Unclear	Unclear	Low	[127]
Kim	Mori ramulus and its Major Component Morusin Inhibit Herpes Simplex Virus Type 1 Replication and the Virus-Induced Reactive Oxygen Species	Low	Unclear	Unclear	Unclear	Low	[103]
Kongyingyoes	3,19-isopropylideneandrographolide suppresses early gene expression of drug-resistant and wild type herpes simplex viruses	Low	Unclear	Unclear	Unclear	Low	[14]
Kumar	Inhibition of herpes simplex virus-1 infection by MBZM-N-IBT: in silico and in vitro studies	Low	Unclear	Unclear	Unclear	Low	[133]
Labrunie	UL23, UL30, and UL5 characterization of HSV1 clinical strains isolated from hematology department patients	Low	Unclear	Unclear	Unclear	Low	[84]
Le-Trilling	Broad and potent antiviral activity of the NAE inhibitor MLN4924	Low	Unclear	Unclear	Unclear	Low	[19]
Lebrun	Varicella-Zoster Virus ORF9p Binding to Cellular Adaptor Protein Complex 1 Is Important for Viral Infectivity	Low	Unclear	Low	Unclear	Low	[139]
Lee	Efficacy of brincidofovir as prophylaxis against HSV and VZV in hematopoietic cell transplant recipients	High	High	High	Unclear	High	[48]
Lei	Preparation of a monoPEGylated derivative of cyanovirin-N and its virucidal effect on acyclovir-resistant strains of herpes simplex virus type 1	Low	High	High	Unclear	Low	[2]
Li	Amentoflavone Inhibits HSV-1 and ACV-Resistant Strain Infection by Suppressing Viral Early Infection	Low	Unclear	Unclear	Unclear	Low	[67]
Li	Anti-herpes simplex virus type 1 activity of Houttuynoid A, a flavonoid from Houttuynia cordata Thunb	Low	Low	Unclear	Unclear	Low	[25]
Liu	Antiviral activities of Janus-type nucleosides and their related oxime-intermediates	Low	Unclear	Unclear	Unclear	Low	[41]
Liu	Harringtonine Inhibits Herpes Simplex Virus Type 1 Infection by Reducing Herpes Virus Entry Mediator Expression	Low	Unclear	Unclear	Unclear	Low	[116]
Lopes	Sulfonated and Carboxymethylated β-Glucan Derivatives with Inhibitory Activity against Herpes and Dengue Viruses	Low	Unclear	Unclear	Unclear	Low	[128]
Luganini	Effective deploying of a novel DHODH inhibitor against herpes simplex type 1 and type 2 replication	Low	Unclear	Unclear	Unclear	Low	[129]
Ma	Herpes simplex virus type 1 (HSV-1) specific T-cell generation from HLA-A1- and HLA-A2-positive donors for adoptive immunotherapy	Low	Unclear	Unclear	Unclear	Low	[12]
Ma	Assessment of a new arbidol derivative against herpes simplex virus II in human cervical epithelial cells and in BALB/c mice	Low	Low	High	Unclear	Low	[75]
Maizel	Study of the Extremely-Tolerant Brevibacterium linens AE038-8 with Antiviral Activity Against Herpes Simplex Virus Type 1	Low	Unclear	Unclear	Unclear	Low	[113]
Mandalari	Simulated human digestion of N1-aryl-2-arylthioacetamidobenzimidazoles and their activity against Herpes-simplex virus 1 in vitro	Low	Unclear	Unclear	Unclear	Low	[72]
Marcocci	The Amphibian Antimicrobial Peptide Temporin B Inhibits In Vitro Herpes Simplex Virus 1 Infection	Low	Unclear	Unclear	Unclear	Low	[54]
Marino-Merlo	Anti-herpes simplex virus 1 and immunomodulatory activities of a poly-γ-glutamic acid from Bacillus horneckiae strain APA of shallow vent origin	Low	Unclear	Unclear	Unclear	Low	[29]
Mello	Perillyl alcohol and perillic acid exert efficient action upon HSV-1maturation and release of infective virus	Low	Unclear	Unclear	Unclear	Low	[92]
Mishra	Herbal Gel Formulation Developed for Anti-Human Immunodeficiency Virus (HIV)-1 Activity Also Inhibits In Vitro HSV-2 Infection	Low	Unclear	Unclear	Unclear	Low	[59]
Mohammed	Synthesis and anti-HSV activity of tricyclic penciclovir and hydroxybutylguanine derivatives	Low	Unclear	Unclear	Unclear	Low	[69]
Monjo	Photodynamic Inactivation of Herpes Simplex Viruses	Low	Unclear	Unclear	Unclear	Low	[55]
Moshaverinia	Evaluation of the effect of hydro alcoholic extract of cinnamon on herpes simplex virus 1	Low	Unclear	Unclear	Unclear	Low	[94]
Musarra-Pizzo	The Antimicrobial and Antiviral Activity of Polyphenols from Almond (Prunus dulcis L.) Skin	Low	Unclear	Unclear	Unclear	Low	[68]
Novoa	Antiviral Activity of Myticin C Peptide from Mussel: an Ancient Defense against Herpes viruses	Low	Unclear	Unclear	Unclear	Low	[8]
Paavilainen	Topical treatment of herpes simplex virus infection with enzymatically created siRNA swarm	Low	Low	High	High	Low	[22]
Parsania	Antiviral screening of four plant extracts against acyclovir resistant herpes simplex virus type-1	Low	Unclear	High	Unclear	Low	[24]
PiresdeMello	Aminomethylnaphthoquinones and HSV-1: in vitro and in silico evaluations of potential antivirals	Low	Unclear	High	Unclear	Low	[3]
Pradhan	Herpes simplex virus virucidal activity of MST-312 and epigallocatechingallate	Low	Unclear	High	Unclear	Low	[63]
Praena	Amidic derivatives of valproic acid, valpromide and valnoctamide, inhibitHSV-1 infection in oligodendrocytes	Low	Unclear	High	Unclear	Low	[78]
Pujol	Polyhydroxylated sulfated steroids derived from 5α-cholestanes as antiviral agents against herpes simplex virus	Low	Unclear	Unclear	Unclear	Low	[16]
Quenelle	Efficacy of pritelivir and acyclovir in the treatment of herpes simplex virus infections in a mouse model of herpes simplex encephalitis	Low	Low	High	Unclear	Low	[35]
Rechenchoski	Mangiferin: A promising natural xanthone from Mangifera indica for the control of acyclovir - resistant herpes simplex virus 1 infection	Low	Low	High	Unclear	Low	[106]
Rittà	Antiviral Activity of a Arisaema Tortuosum Leaf Extract and Some of its Constituents against Herpes Simplex Virus Type 2	Low	Unclear	High	Unclear	Low	[91]
Ruzsics	A Novel, Broad-Acting Peptide Inhibitor of Double-Stranded DNA Virus Gene Expression and Replication	Low	Low	High	Low	Unclear	[90]
Sacchelli	Botryosphaeran and sulfonated derivatives as novel antiviral agents for herpes simplex and dengue fever	Low	Low	High	Low	Unclear	[74]
SadeghEhdaei	Cellular miR-101-1 Reduces Efficiently the Replication of HSV-1 in HeLaCells	Low	Low	High	Unclear	Unclear	[114]
Sanchez	Development and evaluation of a host-targeted antiviral that abrogatesherpes simplex virus replication through modulation of arginine-associated metabolic pathways	Low	Unclear	High	Unclear	Unclear	[11]
Sasaki	In vitro and in vivo antiherpetic effects of(1R,2R)-1-(5'-methylful-3'-yl)propane-1,2,3-triol	Low	Unclear	Unclear	Low	Unclear	[15]
Schneider	Early Steps in Herpes Simplex Virus Infection Blocked by a Proteasome Inhibitor	Low	Low	Low	Unclear	Unclear	[83]
Shabani	Inhibition of herpes simplex virus type 1 replication by novel hsa-miR-7704 in vitro	Low	Unclear	Unclear	Unclear	Unclear	[71]
Shan	Viral UL8 Is Involved in the Antiviral Activity of Oleanolic Acid Against HSV-1 Infection	Low	Unclear	Unclear	Low	Unclear	[130]
Shao	Poly(dA:dT) Suppresses HSV-2 Infection of Human Cervical Epithelial Cells Through RIG-I Activation	Unclear	Unclear	Unclear	Unclear	Unclear	[124]
Sharifi-Rad	Susceptibility of herpes simplex virus type 1 to monoterpenes thymol, carvacrol, p-cymene and essential oils of Sinapis arvensis L., Lallemantiaroyleana Benth. and Pulicaria vulgaris Gaertn	Low	Unclear	Unclear	Low	Unclear	[34]
Sharifi-Rad	Antiviral activity of Veronica persica Poir. on herpes virus infection	Unclear	Unclear	Unclear	Unclear	Unclear	[51]
Shiraki	Helicase-primase inhibitor amenamevir for herpesvirus infection: Towards practical application for treating herpes zoster	Unclear	Unclear	Unclear	Unclear	Unclear	[21]
Shiraki	Amenamevir, a Helicase-Primase Inhibitor, for the Optimal Treatment of Herpes Zoster	Low	Unclear	Low	Low	Unclear	[121]
Spengler	Antiviral, Antimicrobial and Antibiofilm Activity of Selenoesters and Selenoanhydrides	Low	Unclear	Unclear	Low	Unclear	[66]
Stegman	Volatile Acid-Solvent Evaporation (VASE): Molecularly Homogeneous Distribution of Acyclovir in a Bioerodable Polymer Matrix for Long-Term Treatment of Herpes Simplex Virus-1 Infections	Low	Unclear	Unclear	Low	Unclear	[40]
Suryawanshi	Bacterial Pigment Prodigiosin Demonstrates a Unique Anti herpesvirus Activity That Is Mediated through Inhibition of Pro survival Signal Transducers	Low	Unclear	Unclear	Low	Unclear	[109]
Tavakoli	Inhibition of herpes simplex virus type 1 by copper oxide nanoparticles	Low	Unclear	High	Unclear	Unclear	[70]
Tintori	Rhodanine derivatives as potent anti-HIV and anti-HSV microbicides	Low	Unclear	Low	Low	Unclear	[61]
Toscani	Synthesis and Biological Evaluation of Amidinourea Derivatives against Herpes Simplex Viruses	Low	Low	Low	Low	Unclear	[118]
Toulabi	The efficacy of olive leaf extract on healing herpes simplex virus labialis: A randomized double-blind study	Low	Unclear	Unclear	Low	Low	[119]
Tyo	pH-responsive delivery of Griffithsin from electrospun fibers	Low	Unclear	Unclear	Low	Unclear	[80]
Uhlig	Helicase primase inhibitors (HPIs) are efficacious for therapy of human herpes simplex virus (HSV) disease in an infection mouse model	Low	Low	Low	Low	Unclear	[137]
Urbancikova	Efficacy of Pleuran (β-Glucan from Pleurotus ostreatus) in the Management of Herpes Simplex Virus Type 1 Infection	Low	Unclear	Unclear	Low	Unclear	[88]
Vanheule	Basic chemokine-derived glycosaminoglycan binding peptides exert antiviral properties against dengue virus serotype 2, herpes simplex virus-1 and respiratory syncytial virus	Low	Unclear	Unclear	Low	Unclear	[7]
Viegas	Antiviral activity of 1,4-disubstituted-1,2,3-triazoles against HSV-1 in vitro and effects of amino acid changes in drug-resistant α and β herpes viruses DNA polymerase	Low	High	High	Unclear	Unclear	[96]
VilasBoas	Linear antimicrobial peptides with activity against herpes simplex virus 1 and Aichi virus	Unclear	High	High	Unclear	Unclear	[23]
Vilhelmova-Ilieva	Antiviral Activity of Rosa damascena Mill. and Rosa alba L. Essential Oils against the Multiplication of Herpes Simplex Virus Type 1 Strains Sensitive and Resistant to Acyclovir	Unclear	High	High	Low	Low	[131]
Wang	Guanidine modifications enhance the anti-herpes simplex virus activity of (E,E)-4,6-bis(styryl)-pyrimidine derivatives in vitro and in vivo	Low	Unclear	Unclear	Low	Unclear	[108]
Wang	Anti-HSV-1 activity of Aspergilli peptide D, a cyclic pentapepetide isolated from fungus Aspergillus sp. SCSIO 41501	Low	Unclear	Unclear	Low	Unclear	[107]
Whitley	Clinical management of herpes simplex virus infections: past, present, and future	N/A	N/A	N/A	N/A	N/A	[57]
Wright	Inhibition of Herpes Simplex Viruses, Types 1 and 2, by Ginsenoside 20(S)-Rg3	Low	Unclear	Unclear	Low	Low	[105]
Ye	Lupeol impairs herpes simplex virus type 1 replication by inhibiting the promoter activity of the viral immediate early gene α0	Low	Unclear	Unclear	Low	Unclear	[115]
Zhang	NSC23766 and Ehop016 Suppress Herpes Simplex Virus-1 Replication by Inhibiting Rac1 Activity	Low	Unclear	Unclear	Low	Unclear	[117]
Zhou	Anti-HSV-1 effect of dihydromyricetin from Ampelopsis grossedentata via the TLR9-dependent anti-inflammatory pathway	Low	Unclear	Unclear	Low	Unclear	[97]
Zígolo	Chemoenzymatic synthesis of new derivatives of glycyrrhetinic acid with antiviral activity. Molecular docking study	Low	Unclear	Unclear	Low	Unclear	[42]
Zinser	A new promising candidate to overcome drug resistant herpes simplex virus infections	Low	Unclear	High	Low	Unclear	[33]

Principal Findings 

The available literature reviewed consistently supports the existence and potentiality of second-line treatments for HSV strains that are resistant to first-line treatments. We have shown that a majority of the studies reviewed have efficacy as potential managements for resistant strains of HSV [1-3,6-20,22-45,47-52,54-56,58-83,85-106,108-125,127-130,132-138]. The predominance of studies utilized Vero cells [2,3,6,8-10,14-20,23,25,26,28,30-34,36,39-42,44-47,50,51,53-56,58-64,66-70,72,74,76,77,79,82,83,85-87,89-95,97-108,111-113,115-118,120,122,123,125-130,132,133,135,137,138] to test their treatments on. Lastly the most commonly tested treatments that displayed efficacy as second-line treatments in HSV included nectin [17,28,73], amenamevir [21,43,95] and methanol extracts [24,32,39].

Comparison with Existing Literature

This systematic review provides additional information to patients on the potentiality of second-line treatment in HSV strains resistant to first-line treatments. The existing literature comparing medications as treatments for HSV primarily include acyclovir, ganciclovir, and foscarnet [1,2]. There has also been research that compares various plant extracts [24,110]. However, there is a paucity of research that compares greater than 100 second-line HSV interventions for efficacy, as our study has done.

Strengths and limitations

The primary strength of this study is the utilization of the most well-renowned methodology when conducting a systematic review. This included using a prospective protocol created to answer a specific research question, the risk of bias assessment applied to each article, and the summarized table of findings. An additional strength is the relatively large quantity of studies included within this review.

Limitations were that the literature search included language restrictions to only English articles. The search also included interventions for HSV-1, HSV-2, and VZV, and these differences were not considered when assessing efficacy of interventions. Various methodologies were utilized in studies meeting criteria and so the relationship of sample size and efficacy was not able to be successfully analyzed. Another limitation is that the relationship of severity of HSV infections and efficacy of the intervention was not studied.

## Conclusions

Immunocompromised patients have been noted to be the population most affected by drug-resistant variants of HSV. Subsequently, we found that HSV infections within this patient population are challenging to manage clinically effectively. This systematic review provides additional information to patients on the potentiality of second-line treatment in HSV strains resistant to first-line treatments, especially for immunocompromised patients. This review is important as all patients, whether immunocompromised or not, deserve to have their infections clinically managed in a manner supported by comprehensive research. This review provides the necessary information about treatment options for patients with resistant HSV infections and their providers.
